# Probiotic-Enriched Ice Cream with Fermented White Kidney Bean Homogenate: Survival, Antioxidant Activity, and Potential for Future Health Benefits

**DOI:** 10.3390/molecules29133222

**Published:** 2024-07-07

**Authors:** Małgorzata Ziarno, Patrycja Cichońska, Ewa Kowalska, Dorota Zaręba

**Affiliations:** 1 Department of Food Technology and Assessment, Institute of Food Science, Warsaw University of Life Sciences-SGGW (WULS-SGGW), Nowoursynowska 159c St., 02-776 Warsaw, Poland; partycja_cichonska@sggw.edu.pl (P.C.); ewa_kowalska@sggw.edu.pl (E.K.); 2 Professor E. Pijanowski Catering School Complex in Warsaw, 04-110 Warsaw, Poland; dorotazareba@gmail.com

**Keywords:** probiotic ice cream, fermented white kidney bean, antioxidant activity, probiotic survival, gut health, functional ice cream

## Abstract

This study investigated a novel probiotic-enriched ice cream containing fermented white kidney bean homogenate to explore its potential health benefits in the future. We assessed the viability of various probiotic strains during ice cream production and storage, focusing on their potential to reach the gut, and evaluated overall antioxidant activity using 2,2-diphenyl-1-picrylhydrazyl (DPPH), 2,2′-Azino-bis(3-ethylbenzothiazoline-6-sulfonic acid) (ABTS), ferric reducing antioxidant power (FRAP), and total polyphenol content (TPC) assays. The incorporation of fermented white bean homogenate significantly increased antioxidant capacity compared to the control group. Notably, strains such as *Lacticaseibacillus rhamnosus* GG and *Lactiplantibacillus plantarum* 299v demonstrated the most pronounced effects on antioxidant activity, suggesting potential synergistic benefits between probiotics and bioactive compounds in fermented white beans. Although all probiotic strains experienced decreased viability during storage, certain strains, particularly *L. plantarum* 299v and *Lacticaseibacillus casei* DN-114001, showed promising survival rates even after 6 months. These results suggest the potential for developing probiotic ice cream containing viable bacteria capable of reaching the gut and contributing to a healthy gut microbiota. Overall, this study highlights the potential of probiotic-enriched ice cream with fermented white kidney bean homogenate to combine the established benefits of probiotics for gut health with the enjoyment of consuming ice cream.

## 1. Introduction

Traditional ice cream, while undeniably pleasurable, often presents a nutritional paradox due to its high sugar and fat content [1,2], which can limit its overall healthfulness. This observation coincides with the burgeoning popularity of functional foods, reflecting a change in basic assumptions regarding consumer preferences. Consumers are increasingly seeking food products that deliver synergistic benefits, offering both enjoyment and demonstrable health advantages beyond basic nutrition. This trend presents both a challenge and an opportunity for modern food manufacturers. Their task lies in meticulously selecting ingredients that meet the evolving needs of health-conscious consumers while maintaining the sensory appeal that defines their products. Probiotics have emerged as a captivating category of functional food ingredients due to their well-documented health benefits, which include improved gut microbiota composition, enhanced gut barrier function, and modulation of the immune response [3,4]. However, probiotics are sensitive to factors such as freezing and storage conditions, which pose a challenge for their incorporation into frozen desserts like ice cream. Recognizing this potential issue, researchers are actively developing innovative ice cream formulations that incorporate probiotics alongside other health-promoting ingredients. 

This study explores the development of a novel probiotic-enriched ice cream formulation that leverages the potential health benefits of fermented white kidney bean homogenate [5,6]. White kidney beans contain significant quantities of health-promoting compounds, including antioxidant compounds such as polyphenols, as well as proteins [7]. Polyphenols, which are natural defense systems found in plants, possess potent antioxidant properties. By neutralizing free radicals, these antioxidants help mitigate the detrimental effects of oxidative stress, an imbalance at the cellular level linked to chronic diseases. White kidney beans are particularly rich in antioxidant compounds, offering a spectrum of bioactive benefits such as antioxidant, antidiabetic, antiobesity, anti-inflammatory, antimutagenic, and anticarcinogenic properties [8]. Prior to this work, it was known that fermented white kidney beans contain various bioactive compounds, including a wide range of polyphenols, flavonoids, proteins, and specifically isoflavones [9]. These compounds are integral to the health-promoting properties of the beans, improving their nutritional profile and contributing to various biological activities [6]. Gallic acid has been identified as a dominant phenolic compound in kidney beans, with colored beans displaying a higher phenolic content than white ones, although the effects of fermentation on these levels, specifically in white kidney beans, were not detailed [10]. Fermentation, an age-old process, further unlocks the potential of these bioactive compounds by enhancing their bioavailability, thereby potentially amplifying their health-promoting effects [11,12]. By introducing specific microbial strains, fermentation can enhance the bioavailability of bioactive compounds within white kidney beans, unlocking greater health benefits. Bacterial enzymes, in particular, are known to catalyze the transformation of polyphenol molecules, potentially leading to an increase in antioxidant activity [13]. Certain LAB strains have been shown to significantly increase the total antioxidant capacity, as exemplified by the ferric reducing antioxidant power (FRAP) method, thereby underscoring the potential of LAB fermentation to enhance the antioxidant properties of beverages and other functional foods [14].

The antioxidants present in fermented white kidney bean homogenate include various types of antioxidant compounds, such as flavonoids and phenolic acids [7]. These compounds are known for their potent antioxidant properties, aiding in the neutralization of free radicals and reduction in oxidative stress, a factor associated with numerous chronic diseases. However, there is a knowledge gap regarding the impact of incorporating fermented white bean homogenate and probiotic bacteria on the overall functionality of ice cream. While ice cream remains a universally cherished dessert, it presents a unique challenge in incorporating functional ingredients such as probiotics and antioxidants. The key challenges involve ensuring the survival of probiotic bacteria throughout ice cream production and storage, while also maintaining the crucial antioxidant activity of the incorporated compounds. Current research focuses on probiotic ice creams, but there is a significant gap in exploring ice creams enriched with both probiotics and natural antioxidants from fermented plant sources, such as the white kidney bean homogenate used in this study. This innovative approach of integrating fermented white bean homogenate with probiotics in the ice cream matrix holds immense potential. The resulting product could be a true “multifunctional dessert”, offering a symbiotic blend of probiotics and antioxidants. Meanwhile, the retained antioxidant activity from the fermented white bean homogenate can further protect cells from oxidative stress. One concern with using white kidney beans is the presence of natural inhibitors of nutritional enzymes, such as lectins, which can interfere with the digestion and absorption of nutrients. Consuming raw or undercooked white kidney beans can pose risks due to the biologically active lectins, leading to potential health hazards [15]. The fermentation process significantly reduces the levels of these inhibitors, making the homogenate safer for consumption in ice cream [16]. This research aims to bridge the knowledge gap by investigating the viability of probiotic strains throughout ice cream production and storage, while also evaluating the potential for enhanced antioxidant capacity due to the incorporation of fermented white kidney bean homogenate. Through this novel formulation exploration, we aim to contribute to the development of functional ice cream products that offer both indulgence and demonstrable potential health benefits.

This study aimed to comprehensively evaluate the potential for future health-promoting properties associated with probiotic-enriched ice cream containing fermented white kidney bean homogenate. We evaluated the viability of various probiotic strains during ice cream production and storage to ensure their presence and potential nutritional benefits for consumers in the future. Established methods such as 2,2-diphenyl-1-picrylhydrazyl (DPPH), 2,2′-Azino-bis(3-ethylbenzothiazoline-6-sulfonic acid) (ABTS), FRAP, and total polyphenol content (TPC) were employed to assess the overall antioxidant potential of the ice cream containing fermented bean homogenate and probiotic bacteria. These methods provided insights into these products’ ability to neutralize free radicals and potentially mitigate cellular damage. By combining the results on probiotic survival and enhanced antioxidant capacity, we explored the potential for future health benefits associated with consuming this type of ice cream compared to regular ice cream.

## 2. Results and Discussion

### 2.1. Basic Chemical Composition

Our research findings on the basic chemical composition align well with the expected properties of ice cream. The average protein content seems similar across all samples, ranging from 3.00% to 3.03% (Table 1). Similarly, the fat content also appears comparable between the control and other samples, ranging from 5.59% to 5.66%. The dry matter content shows a slightly wider range, with the control sample showing the highest value (26.92%) and the ics1–ics6 samples displaying the lowest (26.60%). However, the error margins are sufficiently large that statistically significant differences may not be evident.

We investigated lactose and its enzymatic breakdown products in the ice cream samples containing milk components. This investigation aimed to identify any changes influenced by bacterial enzymes introduced alongside the fermented white kidney bean homogenate. The lactose content remained consistent across all samples, ranging from 26.71 to 28.36 mg/kg (Table 2). On the other hand, the sucrose content was notably higher, ranging from 110.26 to 116.97 mg/kg. Statistically significant differences were observed between the ics4 sample and the other samples (ics1–ics3, ics5, ics6 samples), indicating potential sucrose decomposition during ice cream production or storage due to enzymatic activity from the microorganisms used (white bean homogenate fermented by *Lacticaseibacillus rhamnosus* GG was utilized in the ics4 samples). Glucose, the third most prevalent sugar, also displayed slight variations among samples, ranging from 4.99 to 5.26 mg/kg. The lowest glucose content was found in the ics2 sample with the addition of white bean homogenate fermented by *Lactobacillus acidophilus* La-5. Other sugars besides lactose, glucose, and sucrose, such as galactose, maltose, raffinose, stachyose, and verbascose, were found in low concentrations, averaging below 0.15 mg/kg. Our interest in carbohydrates for the analysis shown in Table 3 stemmed from the ice cream’s key ingredient: fermented white kidney bean homogenate. As our ice cream formulation combines dairy and bean components, it inherently contains raffinose-series oligosaccharides (RSOs), namely raffinose, stachyose, and verbascose. These oligosaccharides occur naturally in beans and can cause digestive discomfort in some consumers. Their contents were low, with only stachyose showing statistically significant differences between samples fermented with different probiotics. Therefore, we considered the analysis of RSO content crucial for evaluating the overall gut health impact of our product. However, these compounds resist digestion by human enzymes but can be fermented by gut bacteria, resulting in the production of beneficial short-chain fatty acids. Short-chain fatty acids are known for their various health benefits, including improved gut health, reduced inflammation, and enhanced immunity. By analyzing the levels of resistant starches (RSOs), we can assess the potential for digestive issues and guide future product development efforts aimed at mitigating these concerns. The probiotic cultures used in our study have been shown to promote gut health. These probiotics may aid in the breakdown of complex carbohydrates, potentially including some RSOs, thereby reducing digestive discomfort. 

Dry matter, primarily composed of milk solids—not fat and sugars—provides the solid structure of ice cream [17], preventing it from becoming excessively icy or runny. A higher dry matter content results in denser and firmer ice cream. Additionally, it helps trap air bubbles incorporated during churning, creating the light and fluffy texture we all enjoy. Insufficient dry matter would lead to the disappearance of these air bubbles, resulting in a dense, potentially icy product. Although milk protein constitutes a smaller portion of ice cream compared to other ingredients, it plays a crucial role [18]. Milk proteins act like tiny whisks, evenly distributing fat droplets throughout the mixture. Similar to dry matter, protein also aids in stabilizing air bubbles, resulting in a delightful texture. Our research demonstrates the benefit of using white bean homogenate, as it minimally affects protein content, thus contributing to an optimal recipe formulation. While the protein content in our ice cream formulations may not qualify them as a significant source of dietary protein, the inclusion of white beans introduces additional fiber and potentially health-promoting compounds.

Milk fat is another key ingredient that influences both the taste and quality of ice cream [19,20]. During freezing, milk fat crystallizes, forming a network that traps air bubbles and hinders the formation of large ice crystals. Studies have shown that higher fat content results in lower overrun (less air incorporated) [21]. Moreover, the type of fat used affects satiety. Our emphasis on using healthy fats, such as those found in milk and white beans, can offer certain functional properties.

While fat content, sugar levels, and bacterial count do not directly affect ice cream’s acidity, they can influence its pH and viscosity [22]. Our research confirms that yogurt bacteria and probiotic strains generally have minimal impact on the overall carbohydrate content of ice cream. Although they may consume some sugars during fermentation, the amount is negligible compared to the total sugar content. However, probiotics could influence the activity of enzymes that break down carbohydrates, such as alpha-amylase and alpha-glucosidase [23]. The low temperature of ice cream storage significantly hinders bacterial activity. In fact, some research suggests that optimizing sugar concentration might even improve probiotic survival [24]. 

Although there are limited data on the direct effects of probiotics and yogurt bacteria on ice cream’s carbohydrate content, there is potential for the metabolic properties of probiotics to impact the composition and characteristics of dairy products enriched with these microorganisms. Further investigation is necessary to thoroughly understand these effects, especially within the low-temperature environment of ice cream. Changes in fermentative activity could result in reduced levels of unfavorable carbohydrates, such as oligosaccharides that cause flatulence, through breakdown processes. Probiotics, such as *Lactiplantibacillus plantarum*, might influence fermentation in white bean products used in ice cream ingredients, potentially altering carbohydrate content by converting simple and complex sugars [25,26]. Incorporating probiotics into white bean-based products for ice cream formulation could impact their nutritional value, including carbohydrate content, by improving digestibility and potentially stimulating the growth of desirable bacteria that ferment dietary fiber and other carbohydrates [27]. These findings suggest potential health benefits associated with consuming these ice creams, highlighting the need for further investigation.

### 2.2. pH Value

In our experiments, the ice cream recipe incorporating fermented white bean homogenate (samples ics1–ics6) showed a statistically significant decrease in pH compared to the control samples (ics0) containing nonfermented white bean homogenate (Table 3). However, most ice cream samples maintained stable pH values over the 6-month storage period, except for samples ics2 and ics3, which exhibited variations. These observed pH variations might influence the survival of probiotics and the antioxidant properties of the ice cream, topics that will be discussed in the following sections.

The pH value of milk ice cream varies depending on ingredients and production methods, typically ranging from 6.0 to 6.8 [22,28,29,30,31,32]. This range significantly impacts various aspects of ice cream quality. However, there is limited information available on how pH directly affects features such as bacterial survival, overrun, carbohydrate content, or antioxidant properties in ice cream. While some probiotic strains thrive in acidic environments, others struggle [33]. The pH level of fermented ice cream has a significant impact on probiotic survival, with variations depending on the bacterial strains used. For example, *L. acidophilus* thrives in slightly acidic environments (pH 5.5–6.0), whereas bifidobacteria prefer a more neutral range (pH 6.0–7.0). *L. acidophilus* demonstrates better tolerance to acidity compared to bifidobacteria, which experience significantly slowed growth below pH 5.5. Furthermore, even among *Bifidobacterium* species, tolerance to acidity can vary between strains. Therefore, precise pH management during ice cream fermentation is crucial. If the pH drops below 5.5, it can significantly reduce viable probiotic bacteria. 

While pH does not directly affect overrun, it does influence the structure and stability of the ice cream’s protein matrix, impacting its ability to retain air during mixing and freezing. A balanced pH is important for achieving the desired ice cream texture. The direct impact of pH on carbohydrate content is limited. However, pH value can affect the stability of bioactive compounds in ice cream, including their antioxidant properties. Lower pH levels typically increase the antioxidant activity of certain ingredients, but this effect varies depending on the specific compound. pH values can also affect the availability of phenols and other antioxidants in foods.

### 2.3. Ice Cream Overrun 

In determining ice cream texture, volume, and consumer acceptance, overrun plays a crucial role by referring to the amount of air incorporated during churning. Higher overrun results in a lighter texture; however, it can also have a negative effect on probiotic viability. Our experiments showed that the overrun of ice cream was significantly affected by the type of white bean homogenate and the probiotic strain used for fermentation (Table 4). Notably, ice cream made with white bean homogenate fermented with *L. acidophilus* La-5 (ics2) and *L. plantarum* 299v (ics3) exhibited the highest overrun, indicating the significant impact of both the white bean homogenate composition and the bacterial strains employed in fermentation on ice cream overrun. Hydrocolloids, such as starch, possess emulsifying and stabilizing properties that enable them to effectively trap and stabilize air bubbles within the ice cream matrix. The fermentation process, mediated by the bacterial strains used, may enhance the interaction between these hydrocolloids and milk proteins, leading to a more robust and stable air bubble network. Therefore, we hypothesize that the observed variations in ice cream overrun can be attributed to the synergistic effects of white bean homogenate composition and the specific bacterial strains utilized in the fermentation process. The interplay between these factors could modulate the interactions between hydrocolloids and milk proteins, ultimately influencing the formation and stability of air bubbles within the ice cream matrix. To further investigate this hypothesis, future studies could delve into the detailed mechanisms underlying the interactions among hydrocolloids, milk proteins, and the fermentation products generated by the bacterial strains used. Such investigations could provide valuable insights into optimizing the composition and fermentation parameters to achieve the desired ice cream overrun characteristics. 

The observed variations in overrun may also influence the taste and health profile of the ice cream. Ice cream with lower overrun, typically denser due to less air incorporation, might exhibit a more intense flavor profile. Conversely, ice cream with higher overrun could feature a lighter, airier texture that some consumers prefer. From a health perspective, overrun can affect the caloric density of the ice cream. Ice cream with higher overrun will have a lower caloric content per unit volume due to the increased presence of air. However, further investigation is required to fully understand these taste and health implications in the context of white bean fermented ice cream.

While data on overrun specifically for dairy and probiotic ice cream are limited, some general observations can be made. Studies have shown that incorporating plant milk into probiotic ice cream without fermentation can improve sensory aspects and probiotic survival [34]. Additionally, substituting skimmed milk with sweet potatoes in probiotic ice cream has been found to have no significant impact on overrun [35]. Conversely, other research has found no impact of adding probiotics on overrun [36,37]. Further research is necessary to determine the exact mechanisms by which white bean homogenate and lactic acid bacteria influence ice cream overrun. Our findings suggest that overrun is crucial in achieving the desired textural properties of probiotic ice cream while maintaining the viability of probiotic cultures, thereby impacting the potential nutritional value and bioactive properties for consumers. While higher overrun contributes to a lighter texture, it may also strain the probiotics, potentially reducing their survival during processing and storage [38]. Previous research has highlighted the sensitivity of *Bifidobacterium* bacteria to oxygen in dairy products [39,40,41,42]. While further research is required to determine the exact mechanisms, our research supports the idea that ice cream with lower overrun could provide a more conducive environment for probiotic survival [43,44]. Our study aligns with these observations by showing that ice cream with lower overrun levels tended to maintain a higher number of viable probiotic bacteria after processing and storage compared to those with high overrun. Future studies can investigate the interplay between overrun, probiotic strains, and ice cream formulation to optimize both sensory qualities and the potential for future health benefits.

### 2.4. Survival of Yogurt and Probiotic Bacteria

Probiotic ice cream offers a novel approach to delivering beneficial bacteria to the gut, potentially merging enjoyment with probiotic supplementation [34,45,46]. Ensuring probiotic viability throughout processing and storage, while also maintaining sensory and nutritional qualities, is crucial for these functional ice creams.

Table 5 shows the viability (colony-forming units, CFUs) of yogurt and probiotic bacteria strains in ice cream across the production process and during frozen storage. The data are expressed in logarithmic units (log CFUs/mL) for easier comparison. The results of contaminating microflora measurement confirm that the ice cream production process itself does not introduce any contaminating bacteria. While all probiotic strains (ics1–ics6 samples) exhibited a statistically significant decrease in viability throughout the storage period, freezing the ice cream appeared to have a minimal initial impact on the bacterial count for most strains (when comparing values at 0 months). This observation suggests that the chosen probiotic strains possessed some level of tolerance to the freezing process. However, their viability declined over the extended 6-month storage period, emphasizing the need for further investigation into factors influencing long-term probiotic survival in this specific ice cream formulation. The rate of decline varied among strains, as expected due to freezing and storage stress. *Lacticaseibacillus casei* DN-114001 (ics5 samples) displayed the highest resilience, whereas *Lactobacillus delbrueckii* subsp. *bulgaricus* (ics1 samples) showed the most significant decline. These data enable a comparison of the survival of different probiotic strains (ics1–ics6 samples) in ice cream, which is valuable for selecting appropriate strains for ice cream products. Our findings regarding probiotic survival during storage, which generally exceeded the recommended minimum of 6 log CFUs/g, suggest that this ice cream formulation holds promise for delivering viable probiotics to the gut, potentially enhancing health benefits. The observed differences in probiotic survival rates suggest that selecting strains with higher viability after storage (such as *L. plantarum* 299v) could enhance the potential for future health benefits associated with consuming probiotic ice cream.

Although all strains showed a decrease in viability over a 6-month period, certain strains, particularly *L. plantarum* 299v and *L. casei* DN-114001, demonstrated promising survival rates. These findings suggest the potential of probiotic ice cream to deliver viable bacteria to the gut, potentially influencing gut microbiota composition and promoting digestive health, reducing inflammation, and enhancing immune system function—established benefits associated with probiotic consumption. Previous research has shown that probiotics can survive in ice cream for up to 6 months when stored frozen at temperatures ranging from −18 °C to −28 °C, with viable cell counts exceeding the recommended minimum of 6 log CFUs/g [24,30,47]. Factors influencing survival include the strain type, production methods, storage temperature and duration, and the product’s composition (such as bulking agents, sweeteners, and fat content) [33]. Our results corroborate the findings of Salem et al. [44], who observed a decrease in live cell counts for various probiotic strains in ice cream stored at −26 °C. Despite this decline, their ice cream maintained probiotic viability with cell counts above the minimum threshold. Similarly, our study showed a decrease in live cell counts, albeit with different bacterial strains and ice cream formulations, highlighting the influence of both bacterial and ice cream composition on survival rates. Further research involving a wider range of formulations and storage conditions could provide additional insights into how these variables influence probiotic viability.

Digestion can significantly impact the viability of probiotics in fermented food products, including fermented white kidney beans. During the gastrointestinal transit, probiotics must survive the acidic stomach environment and the presence of bile in the small intestine to exert health benefits. Fermented foods, such as white kidney beans, which are enriched with probiotics, may face challenges in maintaining the viability of these beneficial microorganisms throughout the digestive process. However, certain probiotic strains have been identified for their resilience and ability to survive under such harsh conditions, thereby contributing to gut health and offering protective effects against pathogens.

### 2.5. Antioxidant Capacity

**Figure 1:**
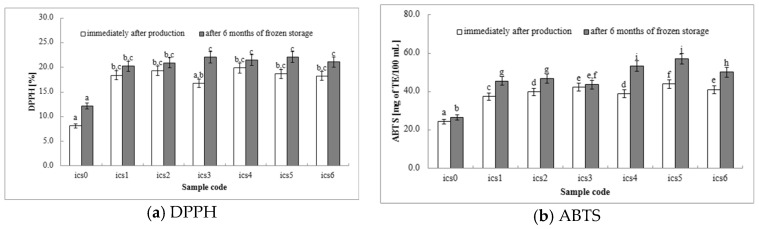

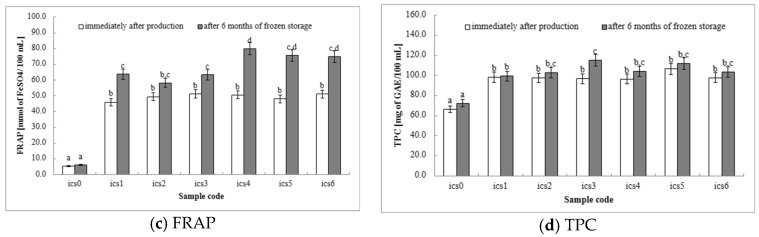
Determination of antioxidant capacity: (**a**) DPPH; (**b**) ABTS; (**c**) FRAP; and (**d**) TPC (total polyphenol content). ^a,b,c,d,e,f,g,h,i^ Same letters indicate a lack of statistically significant difference between the means at the significance level *α* = 0.05.

DPPH: The DPPH assay is a valuable tool for quantifying the antioxidant potential of milk-based ice cream. This assay measures a product’s ability to neutralize free radicals, harmful molecules linked to cellular damage and various diseases. It relies on antioxidants’ ability to scavenge the stable DPPH radical, leading to a reduction in its characteristic purple color. The degree of DPPH radical scavenging directly correlates with the sample’s antioxidant activity, providing a clear indication of the product’s capacity to mitigate oxidative stress. Milk-based ice cream containing fermented white bean homogenate serves as a source of antioxidant compounds, including antioxidant compounds and proteins derived from milk, white beans, and other ingredients. The DPPH assay allows us to assess how effectively these compounds scavenge free radicals. A higher level of antioxidant activity suggests that the ice cream may provide bioactive properties by protecting cells from oxidative stress. All ice cream samples with fermented white bean homogenate (ics1–ics6 samples) exhibited significantly higher DPPH activity compared to the control group (ics0 samples) at both time points (Figure 1a), indicating that fermented white bean homogenate successfully enhances the ice cream’s antioxidant properties. Among the fermented white bean homogenate samples (ics1–ics6 samples), no statistically significant differences in DPPH values were observed either after production or after 6 months of storage, except for the sample containing *L. plantarum* 299v (ics3 samples), where the storage time had a significant effect. Our findings demonstrate that incorporating fermented white bean homogenate with probiotic bacteria into milk-based ice cream represents a promising approach for enhancing its antioxidant potential. This increase in antioxidant activity suggests potential future health benefits, such as reducing cellular damage associated with oxidative stress. Further research could delve into the specific contributions of different bean varieties, fermentation parameters, and storage conditions to the ice cream’s overall antioxidant profile. 

ABTS: The DPPH and ABTS assays measure a product’s ability to neutralize free radicals, which can damage cells and contribute to various diseases. These assays aid in gauging how effective these compounds are in scavenging free radicals. A higher level of antioxidant activity indicates the potential for future health benefits by protecting cells from oxidative stress. ABTS is a common technique for measuring antioxidant activity in various food samples. In the DPPH assay, all ice cream samples containing fermented white bean homogenate (ics1–ics6 samples) exhibited significantly higher ABTS values than the control (ics0 samples) both during production and after 6 months of storage (Figure 1b). This confirms that fermented white bean homogenate effectively enhances the overall antioxidant capacity of the ice cream. Interestingly, the ABTS assay suggests some variability in the impact of different probiotic strains. *L. rhamnosus* GG (ics4 samples) and *L. casei* DN-114001 (ics5 samples) showed the highest values, while *L. plantarum* 299v (ics3 samples) showed a smaller increase. This suggests that these strains may have a more substantial impact on the overall antioxidant profile, warranting further investigation into the specific mechanisms and compounds responsible. Our findings demonstrate that incorporating fermented white bean homogenate with probiotic bacteria presents a promising strategy to improve the antioxidant potential of milk-based ice cream. This elevation in antioxidant activity, as measured by both DPPH and ABTS assays, implies potential health benefits by potentially reducing cellular damage caused by free radicals. Further research could delve into the reasons behind the observed strain-specific differences and identify the specific components contributing to the ice cream’s antioxidant properties. 

FRAP: The FRAP provides an additional method to evaluate the overall antioxidant potential of ice cream, alongside other assays such as DPPH and ABTS. FRAP specifically focuses on the capacity of antioxidant compounds to reduce metal ions, which is a common mechanism through which antioxidants counteract free radicals. As observed in the DPPH and ABTS assays (Section 3.4.5), all ice cream samples containing fermented white bean homogenate (ics1–ics6 samples) exhibited significantly higher FRAP values compared to the control (ics0 samples) both at production and after 6 months of storage (Figure 1c). This confirms that fermented white bean homogenate effectively enhances the ice cream’s overall antioxidant capacity, as measured by all three assays (DPPH, ABTS, and FRAP). Interestingly, the FRAP assay suggests some variation in the impact of different probiotic strains. Except for ics0 and ics2, all samples showed increased FRAP values after storage, with *L. rhamnosus* GG (ics4 samples) showing the most significant increase. This suggests that *L. rhamnosus* GG might have a more significant impact on ferric reducing power, possibly by influencing the types of antioxidant compounds present in the ice cream. However, these findings differ somewhat from the observations related to DPPH values, highlighting the potential for strain-specific effects on various aspects of antioxidant activity. Further investigation is warranted to identify the mechanisms behind these differences and to optimize probiotic selection for maximizing the antioxidant benefits of probiotic ice cream. The heightened ferric reducing power observed with the FRAP assay suggests a potential for these ice creams to enhance antioxidant activity in the body, potentially reducing cellular damage caused by free radicals. 

TPC: The TPC is measured using a colorimetric assay that quantifies the reducing capacity of phenolic compounds, a type of antioxidant found in plants. The increased phenolic content, as measured by TPC, suggests a greater potential for antioxidant activity. Phenolic compounds can function as free radical scavengers, potentially promoting health by reducing oxidative stress in the body. Further research could identify the specific types of phenolic compounds present in the ice cream and determine their individual contributions to the overall antioxidant profile. Figure 1d displays the TPC, expressed in milligrams of gallic acid equivalents (mg GAEs) per 100 mL of ice cream. All ice cream samples containing fermented white bean homogenate (ics1–ics6 samples) exhibited significantly higher TPC values compared to the control group (ics0 samples) both at production and after 6 months of storage. The presence of added probiotic bacteria in the ice cream indicates a greater abundance of phenolic compounds. Conversely, the control group showed minimal changes in TPC, suggesting a low inherent phenolic content. These findings suggest that all yogurt and probiotic strains (ics1–ics6 samples) contribute to the ice cream’s overall phenolic content, possibly due to the presence of phenolics in the fermented white bean homogenate itself or those produced during fermentation. Interestingly, ics3 samples (*L. plantarum* 299v fermentation) showed the most substantial and statistically significant increase in TPC at 6 months. This observation suggests that *L. plantarum* 299v might exert a more significant impact on the overall phenolic content, warranting further investigation into the specific types of phenolics produced and their contribution to antioxidant activity. 

The antioxidant capacity of ice cream is influenced by the presence and concentration of ingredients known for their antioxidant properties. Milk-based ice creams benefit from the inherent nutrients in milk, such as vitamins and proteins, while plant-based options often utilize fruits, nuts, and seeds rich in natural antioxidants, including vitamins and phenolics [34,48,49,50,51]. Our study specifically focused on milk-based ice cream and investigated how incorporating fermented white bean homogenate with probiotic bacteria could enhance its antioxidant capacity compared to regular ice cream. Fermented white bean homogenate is likely to contribute natural antioxidants such as antioxidant compounds (e.g., flavonoids, phenolic acid) and proteins (e.g., albumins, globulins, and phaseolin) [52,53,54,55]. In addition to TPC, fermented white kidney beans are known to contain various other antioxidant compounds, including flavonoids. Quercetin, kaempferol, and apigenin are among the prominent flavonoids found in white kidney beans, renowned for their free radical scavenging properties. Additionally, these beans contain other phenolic acids, with p-coumaric acid and ferulic acid being examples that contribute to their overall antioxidant potential [56,57]. Processes such as soaking, sprouting, and fermentation can influence the antioxidant compounds content of white beans, with fermentation potentially converting complex polyphenols into simpler, more bioavailable forms [11,12]. Studies on the impact of thermal processing and maceration on white beans reveal the presence of kaempferol alongside quercetin and apigenin, supporting the significance of these antioxidant compounds in white kidney beans and their contribution to antioxidant activity [57]. Studies on other legumes, such as soybeans, also suggest that fermentation can influence enzymatic activities and potentially affect polyphenol content [58].

The incorporation of fermented white bean homogenate significantly increased the ice cream’s overall antioxidant activity, as measured by DPPH, ABTS, and FRAP assays. Interestingly, *L. rhamnosus* GG and *L. plantarum* 299v exhibited a more pronounced effect on the ice cream’s antioxidant properties. This suggests a potential synergistic effect between probiotic strains and the bioactive compounds found in fermented white beans. The increased antioxidant activity could offer supplementary bioactive effects by potentially mitigating oxidative stress, which is linked to various chronic diseases. The combination of viable probiotic bacteria and increased antioxidant activity from fermented white beans in this ice cream formulation shows promise for supporting gut health and mitigating oxidative stress. All assays (DPPH, ABTS, FRAP, TPC) demonstrated a significant increase in antioxidant activity in ice cream containing fermented white bean homogenate compared to the control. Fermentation can lead to the formation of bioactive compounds, including plant-derived polyphenols known for their antioxidant properties [59,60,61]. White beans naturally contain antioxidant compounds, and fermentation by lactic acid bacteria or bifidobacteria may release these compounds, potentially increasing the overall antioxidant capacity as observed in fermented soy milk [62,63,64,65]. This suggests a greater ability to neutralize free radicals and potentially protect cells from oxidative stress. 

**Figure 2:**
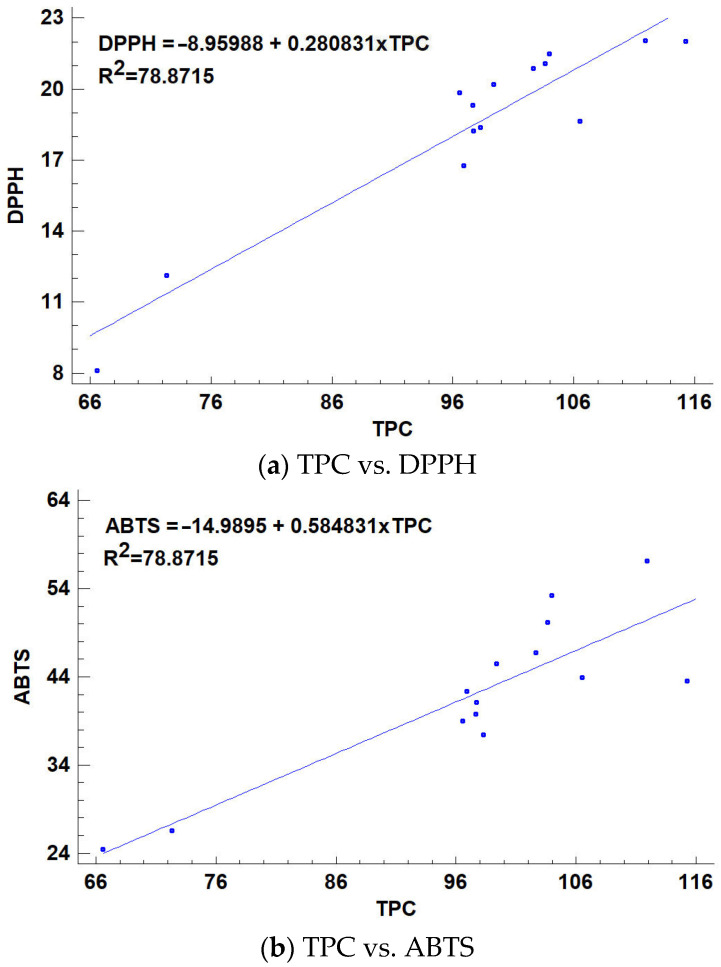

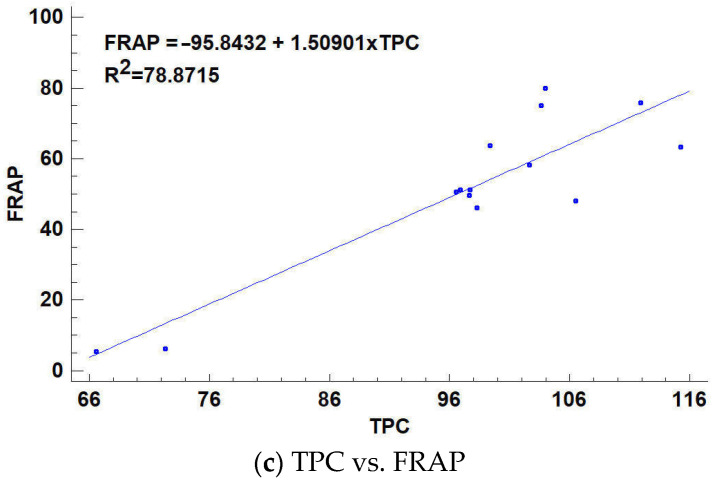
Correlation between total phenolics (TPC) and antioxidant activity (DPPH, ABTS, FRAP) in ice creams before and after 6 months of frozen storage.

The correlation between total phenolics (TPC) and antioxidant activity (measured using DPPH, ABTS, FRAP assays) in ice creams, both before and after 6 months of frozen storage, highlights how storage conditions impact the antioxidant properties and phenolic content (Figure 2). Although there are relatively few specific studies solely focused on TPC and antioxidant activity in ice creams after 6 months of frozen storage, related research offers additional insights. In essence, while phenolic compounds and antioxidant activities are vital aspects of fermented foods, such as milk, their stability and effectiveness may decline with prolonged frozen storage periods. These changes are critical for evaluating the nutritional and health benefits of fermented dairy products, underscoring the need for careful storage practices to preserve their beneficial properties [66,67]. A study focusing on ice cream with significant amounts of pumpkin pulp and carrot pulp showed a valuable content of total phenolics, vitamin C, and antioxidant capacity. This suggests a positive correlation between TPC and antioxidant activity in ice creams enriched with these ingredients. This points toward the potential benefits of incorporating vegetable-based enrichments to maintain antioxidant activity during prolonged storage [68]. While existing studies examine antioxidant activities and total phenolic contents immediately after ice cream production and their potential changes due to in vitro digestion, there is a gap in the literature specifically addressing the effects of long-term frozen storage on these properties. The observed positive impacts shortly after production and postdigestion hint at a potential for preservation during storage; however, the specific results regarding how TPC and antioxidant activities are directly impacted after 6 months of frozen storage await explicit exploration in future studies.

Considering factors that influence antioxidant capacity, such as the specific probiotic strain, fermentation conditions, and bean quality, is important. *L. rhamnosus* GG (ics4 samples) consistently displayed high values across most assays and showed the most substantial increase in FRAP during storage, suggesting a significant impact. Further research is necessary to identify the specific components responsible for this observed increase in antioxidant activity and understand how this process affects the nutritional value of fermented white bean homogenate in ice cream. Generally, storage time resulted in an increase in antioxidant activity as measured by ABTS and FRAP, except for DPPH values. We hypothesize that the observed increase in antioxidant activity may result from enzymatic transformations of polyphenols under freezing conditions. Specifically, bacterial enzymes in the homogenate could be responsible for these changes. The literature extensively documents the capability of bacterial enzymes to modify polyphenol molecules. For instance, various strains of lactic acid bacteria have been shown to possess enzymatic activities that can alter the structure and activity of polyphenols during fermentation and storage [63,64,65]. These enzymatic reactions could potentially enhance the antioxidant properties of the polyphenols, resulting in the observed increase in antioxidant activity during the storage period. Freezing temperatures only slow down the rate of enzymatic reactions but do not stop them, suggesting potential benefits from ongoing fermentation processes even during storage. The overall increase in antioxidant capacity observed in our study suggests potential for future health benefits for consumers, such as reduced cellular damage from free radicals.

Antioxidants are widely recognized for their health benefits. However, there is growing evidence that excessive consumption of antioxidants can lead to undesirable health effects. Research suggests that elevated levels of antioxidants in the body can not only neutralize free radicals but also influence various biological processes that may promote cancer development. A seminal study in this context is the work by Kadosh et al. [69], which showed how the intestinal microbiome can transform the p53 protein mutant from a suppressor to an oncogenic function. High concentrations of antioxidants might affect the interactions between the microbiome and the p53 protein, leading to unfavorable changes. The authors concluded that the presence of antioxidants in the intestine could promote tumor development through this transformation. Another important study by Sayin et al. [70] showed that antioxidants can accelerate the progression of lung cancer in mice. This study indicates that while antioxidants can protect cells from damage, excessive antioxidants can promote the growth of existing cancer cells. This finding suggests that controlling antioxidant levels is crucial to avoid potential risks. In summary, although antioxidants play a key role in protecting cells from oxidative stress and related diseases, excessive consumption of antioxidants can lead to undesirable health effects. For this reason, it is important to aim for a moderate intake of antioxidants and avoid supplementation without consulting a doctor. Appropriate research on the health effects of antioxidants should continue to better understand their effects and ensure their safe use in the diet. In the context of our research, we emphasize the need for further analysis on the impact of increased antioxidant capacity in ice cream with the addition of fermented white bean homogenate on consumer health.

Digestion significantly impacts the antioxidant activity of fermented food products, including fermented white kidney beans. The bioavailability and efficacy of antioxidants can be either enhanced or reduced during digestion, depending on factors such as the chemical structure of the antioxidants, the composition of the food matrix, and the fermentation products. Fermentation can lead to the breakdown of complex compounds into simpler, more absorbable forms, potentially increasing antioxidant activity by releasing bound phenolic compounds and enhancing their absorption and bioavailability. Research suggests that fermentation can increase the content of bioactive compounds, such as polyphenols, in legumes, thus improving their antioxidant potential. The bioconversion processes during fermentation can generate new bioactive compounds or increase the free form of existing antioxidants, which may exhibit higher antioxidant activity than their precursor compounds. However, a thorough investigation into the impact of digestion on these properties requires targeted research examining how probiotic strains and specific antioxidant compounds in fermented white kidney beans interact and undergo modifications throughout the gastrointestinal tract.

## 3. Materials and Methods

### 3.1. Materials

The biological materials used in this study comprised a commercial yogurt starter culture, YC-180 (Chr. Hansen, containing *Streptococcus thermophilus*, L. *delbrueckii* subsp. *bulgaricus*), along with five probiotic strains: *L. acidophilus* La-5, *L. plantarum* 299v, *L. rhamnosus* GG, *L. casei* DN-114001, and *Bifidobacterium animalis* subsp. *lactis* Bb-12. These materials were all deposited in the institute’s collection. 

The ice cream samples were prepared using the following ingredients: 215 g of pasteurized milk with a 3.2% fat content (OSM Łowicz, Poland), 45 g of Ultra-High Temperature (UHT) cream with a 36% fat content (OSM Łowicz, Poland), 50 g of granulated sugar (Diamant Poland), 105 g of white bean homogenate (as specified in Table 6), 5 mL of a suspension of starter culture YC-180 or a suspension of cultured and concentrated biomass of live probiotic strain cells, and 0.5 mL of vanilla flavoring (Delecta Trade, Poland). The white kidney beans of the “Piękny Jaś Karłowy” (*Phaseolus vulgaris*) variety were procured from a local market.

### 3.2. Dairy–Fermented Bean Ice Cream 

The ice cream recipe comprised the following ingredients by weight percentage: pasteurized milk (3.2% fat content) at 51.1%, UHT cream (36% fat content) at 10.7%, granulated beetroot sugar at 11.9%, white bean homogenate at 25.0%, bacterial biomass at 1.2%, and vanilla flavor at 0.1%. 

The white bean homogenate preparation and fermentation followed a previously established laboratory method [25]. One hundred grams of whole, healthy beans underwent germination in a kitchen germinator at 25 ± 0.5 °C for 72 h, with regular moistening using tap water every 24 h. After germination, the beans, along with the absorbed water, were transferred to a larger container filled with water, resulting in a final weight of 500 g for the bean–water mixture, indicating 400 g of water was added to the germinated beans. The mixture was then blended in a kitchen blender for 20 min at room temperature to create a homogenate. This homogenate was brought to a boil and continuously stirred to gelatinize the bean starch, aiding in the breakdown of complex carbohydrates for potential accessibility to probiotic bacteria during fermentation. Subsequently, the beverage underwent filtration through a 0.1 mm sieve to remove any remaining bean particles. The filtered homogenate was collected in a clean container and sterilized at 121 °C for 20 min to eliminate potential contaminating microorganisms and neutralize very toxic compounds such as phytohemagglutinin [15]. Following sterilization, the homogenate was cooled down to 45 ± 0.5 °C, a suitable temperature for the growth of the probiotic cultures used in the fermentation process. 

Before being used in the fermentation process, the probiotic strains underwent revival and propagation in MRS broth (for lactobacilli) or in BSM broth (for bifidobacteria) aliquots at 37 ± 0.5 °C for 18 h. An 18 h broth culture was then subjected to centrifugation in an ultracentrifuge (model type 317a, Precision Mechanics Warsaw, Poland) for 5 min at 12,000× *g* at 4 ± 0.5 °C. The deep plate method (MRS agar for lactobacilli or BSM agar for bifidobacteria, 37 ± 0.5 °C, 72 h, anaerobic conditions) was employed to determine the number of viable bacterial cells in the culture broth. Following this, the sterilized white bean homogenate was inoculated with 6 CFUs/mL (colony-forming units per milliliter) of probiotic strain culture. The starter culture YC-180 was applied as per the manufacturer’s instructions, after reconstitution in sterile water. No additional nutrients were added to the homogenate. The white bean homogenate samples were stirred and maintained at 43 ± 0.5 °C for 5 h to undergo the fermentation process. The pH of the fermenting mixture was periodically monitored (e.g., every hour) to track fermentation progress [25]. Subsequently, the samples were cooled to 4 ± 0.5 °C to lower the temperature and stop the fermentation process. 

**Figure 3:**
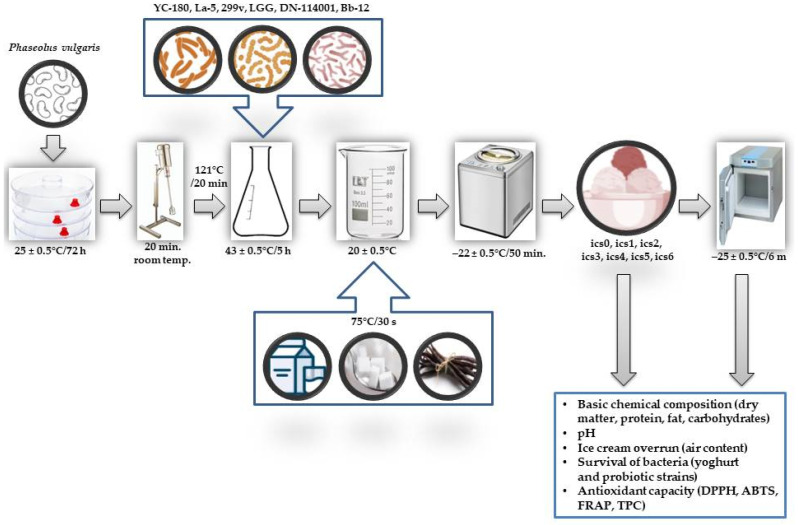
Experimental process: a visual overview.

The ice cream mixture underwent homogenization using a Kenwood HB714P hand blender (Kenwood Poland, Warsaw, Poland). Following this, the ice cream base (excluding the white bean homogenate) underwent pasteurization (75 °C/30 s) and was cooled to 20 ± 0.5 °C before incorporating the white bean homogenate. Subsequently, the ice cream mixture was poured into an Unold type 8875 ice cream maker (Unold Poland, Gdynia, Poland). The ice cream production process lasted 50 min and involved freezing the mixture to a temperature of −22 ± 0.5 °C. The experiment was performed in three independent repetitions. All ice cream samples were analyzed immediately after freezing and again after 1, 2, 3, 4, 5, and 6 months of storage at −25 ± 0.5 °C (Figure 3).

### 3.3. Media and Other Reagents

The MRS broth, MRS agar, M17 agar, BSM broth, BSM agar, VRBG, and YGC were prepared in the appropriate quantities according to the manufacturer’s instructions (Merck, Darmstadt, Germany). All reagents required for the analytical methods described below were obtained from local chemical reagent distributors and met analytical-grade standards. 

### 3.4. Methods

#### 3.4.1. Basic Chemical Composition

Dry matter (DM) determination: The samples were oven-dried at 102 ± 0.5 °C until a constant weight was achieved, typically within 3 h [25]. All analyses were performed in triplicate and reported to two decimal places.

Determination of protein content: The Kjeldahl method was used to determine the protein content, with crude protein content calculated as *N* × 6.25 [25]. All analyses were performed in triplicate and reported to two decimal places.

Determination of fat content: The Gerber method was utilized to determine fat content, with all analyses performed in triplicate and reported to two decimal places.

Determination of contents of selected carbohydrates (lactose, glucose, galactose, sucrose, and RSO): High-performance liquid chromatography (HPLC) was employed for carbohydrate analysis, following the protocol described in our previous publications [25,26,71], with slight modifications. An HPLC system was utilized for chromatographic analysis, consisting of the following components: DeltaChrom Pump Injector (S6020 Needle Injection Valve, Sykam, Eresing, Germany), DeltaChrom Temperature Control Unit (Sykam, Germany), refractive index detector (S3580 RI Detector, Sykam, Germany), precolumn Guard Column Sugar-D (10 mm × 4.6 mm, 5 µm; Cosmosil, Nacalai Tesque, Kyoto, Japan), and Column Sugar-D (250 mm × 4.6 mm, 5 µm; Cosmosil). The separation parameters for HPLC analysis were set as follows: flow rate: 1 mL/min, oven temperature: 30 ± 0.1 °C, detector range: 10,000 mV, sampling rate: 2 Hz. The mobile phase employed was a 60:40 (*v*/*v*) mixture of HPLC-grade acetonitrile (Sigma-Aldrich, St. Louis, MO, USA) and deionized water. Each analysis was completed within approximately 40 min using an isocratic separation. All analyses were conducted in triplicate and reported to two decimal places.

#### 3.4.2. pH Measurement

The pH of the ice cream samples was measured using an Elmetron CPO-505 pH meter (Elmetron, Zabrze, Poland). The samples were initially thawed at 18 °C for 15 min, and the pH reading was recorded after approximately 30 s, once the value had stabilized. All analyses were performed in triplicate and reported to two decimal places. 

#### 3.4.3. Ice Cream Overrun

The ice cream overrun is determined by measuring the volume of air within a specified volume of ice cream sample. This air volume is calculated by subtracting the volume of the ice cream sample from the volume obtained after melting the ice cream. The overrun is then expressed as the percentage ratio of the air volume to the volume of the melted ice cream mixture. 

One day before performing the ice cream overrun determination, cylindrical metal containers with a volume of 150 mL were placed in a freezer (−30 ± 0.5 °C) to thoroughly chill. Subsequently, the containers were carefully filled with ice cream samples, ensuring no empty spaces, air bubbles, or pressing down on the ice cream. Using a funnel, the entire ice cream sample was then transferred from the container to a 250 mL measuring flask. Once the ice cream had completely melted, the measuring flask was filled to the mark using distilled water measured from a burette. All analyses were performed in triplicate and reported to two decimal places. The ice cream overrun was calculated according to Formula (1):

(1) $$P=\frac{a-(V1-V2)}{V1-V2}\times 100\;[\%],$$

where *a* represents the volume of the ice cream sample [mL], which is 150 mL; *V*1 represents the capacity of the measuring flask [mL], which is 250 mL; *V*2 represents the volume of added distilled water [mL]; and *P* represents the ice cream overrun [%]. 

#### 3.4.4. Determination of the Survival of Yogurt and Probiotic Bacteria

The total viable counts of yogurt bacteria and probiotic strains were enumerated using the plate technique [1]. De Man, Rogosa, and Sharpe (MRS) agar (Merck, Darmstadt, Germany) was used to determine the counts of lactobacilli, M17 agar (Merck) for the counts of streptococci, and BSM agar (Sigma-Aldrich) without additional supplementation for the counts of bifidobacteria. The plates inoculated with diluted samples were then incubated at 37 ± 0.5 °C for 72 h under either aerobic conditions (for streptococci) or anaerobic conditions (for lactobacilli and bifidobacteria) using Anaerocult (Merck). Following incubation, CFUs per gram of the original beverage sample were determined by counting colonies and expressing the results as log CFUs/g. All analyses were performed in triplicate and reported to two decimal places.

Alongside measuring the counts of starter culture bacteria, we also investigated the presence of contaminating microflora. The total number of Enterobacteriaceae was determined in VRBG medium (with crystal violet, neutral red, bile salts, and glucose, Merck) overlaid on the same medium and then incubated at 37 °C for 24 h. Additionally, the total numbers of molds and yeasts were determined in YGC medium (yeast extract glucose chloramphenicol agar FIL-IDF, Merck) with plates incubated at 25 °C for 5 days. The results were expressed in colony-forming units per gram.

#### 3.4.5. Determination of Antioxidant Capacity

DPPH: To conduct this assay, 0.2 mM 1,1-diphenyl-2-picrylhydrazyl (DPPH) in ethanol (0.8 mL) was mixed with 0.2 mL aliquots of each sample at varying concentrations (ranging from 100 to 5000 μg/mL). After the resulting mixtures were vortexed, they were incubated in darkness for 10 min at 25 ± 0.5 °C. Subsequently, the absorbance of the samples was measured at 492 nm using a Helios Gamma UV–Vis spectrophotometer (Thermo Fisher Scientific, Waltham, MA, USA). All analyses were performed in triplicate, and the results were reported to two decimal places. The percentage of DPPH radical scavenged (RSA) was calculated using the following Formula (2):

(2) $$RSA=\frac{1-A1}{A0}\times 100\;[\%],$$

where *A*1 represents the absorbance of the sample solution at 492 nm, *A*0 represents the absorbance of the blank solution (0.8 mL ethanol added to 0.2 mL of sample solution), and RSA indicates the Radical Scavenging Activity [%]. 

ABTS: In this study, the total antioxidant capacity of ice cream samples was assessed using the ABTS method as described by [72], with minor modifications. Our aim was to quantify the ice cream’s overall ability to scavenge free radicals, particularly in ice cream containing fermented white bean homogenate and probiotic bacteria. To prepare the ABTS radical stock solution, potassium persulfate (2.6 mM, Sigma-Aldrich) was added to an aqueous ABTS solution (7 mM, Sigma-Aldrich). This mixture was stored in the dark at room temperature for 12–16 h. Subsequently, the stock solution was diluted with methanol (POCH, Gliwice, Poland) to achieve a final absorbance of approximately 1.1 ± 0.02 at 734 nm, forming the working solution. Ice cream samples (0.3 mL) were then combined with the working solution (2.7 mL) and allowed to incubate at room temperature for 30 min. After incubation, the mixtures underwent centrifugation at 12,000× *g* for 2 min at 20 ± 0.5 °C. The decrease in absorbance at 734 nm was measured against a methanol blank using a Helios Gamma UV–Vis spectrophotometer (Thermo Fisher Scientific). All analyses were performed in triplicate, and the results were reported to two decimal places. The results were expressed as Trolox equivalent (TE) antioxidant capacity (TEAC) per 100 mL of ice cream samples, with Trolox^®^ (Sigma-Aldrich) serving as the reference standard.

FRAP: The FRAP assay, originally described by [73], was employed to evaluate the antioxidant capacity of the samples. This method relies on the reduction of a tripyridyltriazine (TPTZ) complex, resulting in a color change when antioxidants are present. A fresh FRAP reagent was prepared by mixing 5 mL of 10 mmol/L TPTZ solution in 40 mmol/L HCl with 5 mL of 20 mmol/L FeCl_3_ solution and 50 mL of 0.3 mol/L acetate buffer, maintaining it at 37 ± 0.5 °C. Aliquots of sample supernatant (50 μL) were mixed with 1.5 mL of FRAP reagent and incubated at 37 ± 0.5 °C for 10 min. The absorbance of the resulting mixture was measured at 593 nm using a spectrophotometer. A calibration curve was generated using eight FeSO_4_ × 7H_2_O concentrations (ranging from 100 to 1000 mmol/L). The absorbance of the calibration standards was measured similarly to the samples. The results were expressed as antioxidant concentration per 100 mL of ice cream samples, with ferric reducing capacity equivalent to 1 mmol/L FeSO_4_ [73]. All analyses were conducted in triplicate and reported to two decimal places.

Total polyphenol content (TPC) using the Folin–Ciocalteu method: TPC provides a measurement of the overall phenolic content, which can enhance the ice cream’s antioxidant properties. In addition, TPC values can indicate whether the fermented white bean homogenate and probiotic bacteria contribute to the ice cream’s phenolic content. To create a calibration curve, a standard solution of gallic acid (Pol–Aura, Poland, with a reagent purity of ≥99%) was used. Calibration solutions of the acid were prepared in 25 mL measuring flasks with concentrations ranging from 0 to 2.5 mg/mL in increments of 0.5 mg/mL. Absorbance was measured at a wavelength of 765 nm against a blank (0 mg/mL gallic acid solution) using plastic cuvettes (Bionovo Poland, Legnica, Poland) and a Helios Gamma UV–Vis spectrophotometer (Thermo Fisher Scientific). Based on the measured absorbances, a calibration curve was plotted and utilized to calculate the TPC in the analyzed ice cream samples in terms of gallic acid equivalents (GAEs). Once the calibration curve was established, the absorbance of the ice cream samples’ test solutions was measured. Thawed ice cream samples (thawed at 18 ± 0.5 °C for 30 min) were portioned into 30 g amounts in Falcon tubes, capped, and centrifuged using an MPW-350 laboratory centrifuge (MPW Med. Instruments, Spółdzielnia Pracy, Warsaw, Poland) at 10,000× *g* for 20 min at 4 ± 0.5 °C. Following centrifugation, the tubes exhibited three layers: oil at the top, supernatant in the middle, and sediment at the bottom. 0.25 mL of the supernatant layer was withdrawn and transferred to 25 mL measuring flasks. Subsequently, 15 mL of distilled water and 1.25 mL of Folin–Ciocalteu reagent were added, mixed, and allowed to stand for 3 min at room temperature. Then, 3.75 mL of saturated sodium carbonate solution was added, the solution was topped up with distilled water, mixed, and placed in a 40 °C thermostat for 30 min before the absorbance at a wavelength of 765 nm was measured. The TPC in the ice cream was calculated based on the calibration curve and expressed in terms of GAEs. All analyses were performed in triplicate and reported to two decimal places, with results expressed as GAEs per 100 mL of ice cream samples.

### 3.5. Statistical Analysis

The entire experiment was conducted in three independent replicates, with each analysis performed in two replicates for each experiment, resulting in an average of nine replicates per result. The data underwent a two-way analysis of variance, and mean differences among the statistical groups were assessed at a significance level of *α* = 0.05 using Tukey’s test within the ANOVA method. Statistical analysis of the results was carried out using the Statgraphics Centurion XVII v. 17.2.00 program from Statgraphics Technologies Inc., based in The Plains, VA, USA.

## 4. Conclusions

This study investigated the viability of probiotic bacteria in ice cream enriched with fermented white bean homogenate during storage. The ice cream formulations exhibited minor variations in basic chemical composition, maintaining consistent protein and fat contents across all samples. The inclusion of fermented white bean homogenate resulted in a significant decrease in pH compared to controls, affecting probiotic survival and antioxidant properties. pH stability during storage varied, which influenced the overall quality and health-promoting potential of the ice cream. Overrun values varied significantly based on the type of white bean homogenate and probiotic strain used, affecting both texture and probiotic viability. Understanding these factors is critical for optimizing product attributes and ensuring consumer acceptance. Differences in sugar content, particularly for sucrose, were observed due to enzymatic activity from probiotic fermentation, highlighting potential impacts on product formulation and health considerations. Probiotic strains exhibited varying degrees of viability during storage, with notable resilience observed in some strains despite freezing stress. This underscores the importance of selecting appropriate strains and maintaining suitable storage conditions to preserve probiotic efficacy in functional ice cream products. Furthermore, fermented white bean homogenate enhanced the ice cream’s antioxidant activity, as demonstrated by increased DPPH, ABTS, and FRAP values and total phenolic content. These findings suggest potential health benefits, such as reduced oxidative stress, associated with consuming probiotic-enriched ice cream. 

In conclusion, integrating fermented white kidney bean homogenate into probiotic-enriched ice cream presents a promising strategy to enhance its nutritional profile and potential health benefits. Future research should focus on optimizing formulation parameters and evaluating long-term consumer health outcomes to fully realize the benefits of these innovative ice cream products. While this study demonstrates the potential functional properties of probiotic ice cream containing fermented white bean homogenate, further research is necessary to fully understand the specific health effects and determine the optimal dosage for consumption. Optimizing probiotic survival during storage and identifying strains with maximized viability and contributions to antioxidant activity are crucial for maximizing the potential for future health benefits. Finally, exploring the sensory attributes and consumer acceptance of this functional ice cream is essential for its successful commercialization. Additionally, we recognize the value of understanding the physical properties of our ice cream formulation. Characterizing the physical properties of this novel ice cream is important for future research, which could involve analyses of factors such as texture, melting profile, and overrun. 

While antioxidants play a beneficial role in protecting against oxidative stress, excessive intake may lead to unintended health consequences, potentially influencing biological processes involved in cancer development. This underscores the importance of moderation in antioxidant consumption and emphasizes the need for further research into their effects. The process of digestion significantly influences the antioxidant activity of fermented foods, such as fermented white kidney beans. Fermentation enhances bioavailability by releasing bound phenolic compounds and potentially forming new bioactive compounds with higher antioxidant activity. However, the specific impacts of digestion on these properties necessitate further investigation, particularly regarding the interplay of probiotic strains and antioxidant compounds in fermented white kidney beans. Future research efforts should prioritize optimizing formulation strategies and comprehensively understanding the digestive impacts to ensure the safe and effective utilization of these functional food products.

## Figures and Tables

**Table 1:** Basic chemical composition of ice cream obtained.

Sample Code	Proteins [%]	Fat [%]	DM [%]
Control sample	3.03 ^a^ ± 0.15	5.66 ^a^ ± 0.26	26.92 ^a^ ± 1.14
ics1 samples	3.03 ^a^ ± 0.11	5.56 ^a^ ± 0.20	26.58 ^a^ ± 0. 98
ics2 samples	2.97 ^a^ ± 0.14	5.61 ^a^ ± 0.20	26.59 ^a^ ± 1.13
ics3 samples	2.98 ^a^ ± 0.12	5.59 ^a^ ± 0.22	26.60 ^a^ ± 0.99
ics4 samples	3.00 ^a^ ± 0.13	5.59 ^a^ ± 0.21	26.57 ^a^ ± 1.04
ics5 samples	3.01 ^a^ ± 0.10	5.56 ^a^ ± 0.23	26.61^a^ ± 0.99
ics6 samples	2.98 ^a^ ± 0.12	5.58 ^a^ ± 0.22	26.62 ^a^ ± 0.96

Legend: ^a^ Same letters in the same column indicate a lack of statistically significant difference between the means at the significance level *α* = 0.05; DM: dry matter.

**Table 2:** Selected carbohydrates in analyzed ice cream samples.

Sample Code	Average Content of Carbohydrates Assessed [mg/kg]								
	Lactose	Glucose	Galactose	Fructose	Sucrose	Maltose	Raffinose	Stachyose	Verbascose
ics0	28.27 ^a^ ± 1.55	5.26 ^b^ ± 0.29	0.00 ^a^ ± 0.00	0.01 ^a^ ± 0.00	116.23 ^a,b^ ± 3.00	0.04 ^a^ ± 0.00	0.02 ^a^ ± 0.00	0.14 ^c^ ± 0.01	0.01 ^a^ ± 0.00
ics1	26.79 ^a^ ± 1.14	5.23 ^b^ ± 0.27	0.00 ^a^ ± 0.00	0.01 ^a^ ± 0.00	115.63 ^b^ ± 2.45	0.04 ^a^ ± 0.00	0.02 ^a^ ± 0.00	0.14 ^c^ ± 0.01	0.01 ^a^ ± 0.00
ics2	28.09 ^a^ ± 1.54	4.99 ^a^ ± 0.28	0.00 ^a^ ± 0.00	0.01 ^a^ ± 0.00	116.97 ^b^ ± 2.41	0.04 ^a^ ± 0.00	0.02 ^a^ ± 0.00	0.13 ^b,c^ ± 0.01	0.01 ^a^ ± 0.00
ics3	28.36 ^a^ ± 1.47	5.23 ^b^ ± 0.27	0.00 ^a^ ± 0.00	0.01 ^a^ ± 0.00	116.97 ^b^ ± 1.17	0.04 ^a^ ± 0.00	0.02 ^a^ ± 0.00	0.12 ^a,b^ ± 0.01	0.01 ^a^ ± 0.00
ics4	27.02 ^a^ ± 1.36	5.26 ^b^ ± 0.20	0.00 ^a^ ± 0.00	0.01 ^a^ ± 0.00	110.26 ^a^ ± 3.14	0.04 ^a^ ± 0.00	0.02 ^a^ ± 0.00	0.12 ^a,b^ ± 0.01	0.01 ^a^ ± 0.00
ics5	28.26 ^a^ ± 1.51	5.00 ^a,b^ ± 0.19	0.00 ^a^ ± 0.00	0.01 ^a^ ± 0.00	116.97 ^b^ ± 2.88	0.04 ^a^ ± 0.00	0.02 ^a^ ± 0.00	0.11 ^a^ ± 0.01	0.01 ^a^ ± 0.00
ics6	26.71 ^a^ ± 1.44	5.26 ^b^ ± 0.22	0.00 ^a^ ± 0.00	0.01 ^a^ ± 0.00	116.36 ^a,b^ ± 3.01	0.04 ^a^ ± 0.00	0.02 ^a^ ± 0.00	0.11 ^a^ ± 0.01	0.01 ^a^ ± 0.00

Legend: ^a,b,c^ Same letters in the same column indicate a lack of statistically significant difference between the means at the significance level *α* = 0.05.

**Table 3:** Ice cream pH immediately after production and after 6 months of frozen storage.

Sample Code	Sampling Time [Month]	
	0	6
ics0	6.60 ^a^ ± 0.16	6.55 ^a^ ± 0.15
ics1	5.61 ^c,d^ ± 0.13	5.57 ^d^ ± 0.13
ics2	5.87 ^b^ ± 0.18	5.63 ^d^ ± 0.13
ics3	5.82 ^b,c^ ± 0.20	5.54 ^d^ ± 0.10
ics4	5.61 ^c,d^ ± 0.30	5.49 ^d^ ± 0.13
ics5	5.95 ^b^ ± 0.15	5.88 ^b^ ± 0.10
ics6	5.74 ^c^ ± 0.14	5.66 ^c,d^ ± 0.11

Legend: ^a,b,c,d^ Same letters in the whole table indicate a lack of statistically significant difference between the means at the significance level *α* = 0.05.

**Table 4:** Ice cream overrun [%] immediately after production and after 6 months of frozen storage.

Sample Code	Sampling Time [Month]	
	0	6
ics0	15.42 ^b^ ± 0.11	15.10 ^a,b^ ± 0.15
ics1	14.70 ^a^ ± 0.44	14.67 ^a^ ± 0.47
ics2	18.23 ^d^ ± 0.59	18.13 ^d^ ± 0.59
ics3	17.47 ^c,d^ ± 0.55	17.37 ^c,d^ ± 0.65
ics4	15.43 ^a,b,c^ ± 0.49	15.33 ^a,b,c^ ± 0.49
ics5	16.83 ^c^ ± 0.50	16.80 ^c^ ± 0.53
ics6	15.17 ^a,b^ ± 0.47	15.10 ^a,b^ ± 0.54

Legend: ^a,b,c,d^ Same letters in the whole table indicate a lack of statistically significant difference between the means at the significance level *α* = 0.05.

**Table 5:** Survival of yogurt and probiotic bacteria cells (expressed as log CFUs/mL) in ice cream before and after its production and during 6 months of frozen storage.

Sample Code/Population		Sampling Time [Month]							
		0 (before Freezing)	0 (after Freezing)	1	2	3	4	5	6
ics0	No bacteria added	0.0 ^a^ ± 0.0	0.0 ^a^ ± 0.0	0.0 ^a^ ± 0.0	0.0 ^a^ ± 0.0	0.0 ^a^ ± 0.0	0.0 ^a^ ± 0.0	0.0 ^a^ ± 0.0	0.0 ^a^ ± 0.0
ics1	*S. thermophilus*	8.7 ^b^ ± 0.2	8.7 ^b^ ± 0.2	8.7 ^b^ ± 0.2	8.6 ^a,b^ ± 0.3	8.6 ^a,b^ ± 0.3	8.5 ^a^ ± 0.3	8.4 ^a^ ± 0.2	8.4 ^a^ ± 0.3
	*L. delbrueckii* subsp. *bulgaricus*	6.8 ^c^ ± 0.2	6.8 ^c^ ± 0.2	6.4 ^b,c^ ± 0.4	6.2 ^a,b^ ± 0.2	6.2 ^a,b^ ±0.2	6.2 ^a,b^ ± 0.2	6.1 ^a^ ± 0.3	5.9 ^a^ ± 0.2
ics2	*L. acidophilus* La-5	8.7 ^e^ ± 0.2	8.6 ^d,e^ ± 0.2	8.5 ^d^ ± 0.3	8.4 ^c,d^ ± 0.3	8.3 ^c^ ± 0.2	8.2 ^c^ ± 0.3	7.4 ^b^ ± 0.2	7.1 ^a^ ± 0.2
ics3	*L. plantarum* 299v	8.6 ^d^ ± 0.2	8.7 ^d^ ± 0.2	8.3 ^c^ ± 0.3	8.2 ^c^ ± 0.3	8.1 ^c^ ± 0.3	7.5 ^b^ ± 0.2	7.3 ^b^ ± 0.2	6.7 ^a^ ± 0.2
ics4	*L. rhamnosus* GG	8.7 ^c^ ± 0.2	8.7 ^c^ ± 0.1	8.5 ^b,c^ ± 0.2	8.4 ^b^ ± 0.2	8.2 ^b^ ± 0.3	8.2 ^b^ ± 0.2	7.3 ^a^ ± 0.2	7.0 ^a^ ± 0.2
ics5	*L. casei* DN-114001	8.9 ^d^ ± 0.1	8.8 ^d^ ± 0.1	8.6 ^c^ ± 0.1	7.7 ^b,c^ ± 0.2	7.4 ^b^ ± 0.1	7.5 ^b^ ± 0.3	7.4 ^b^ ± 0.3	6.7 ^a^ ± 0.2
ics6	*B. animalis* subsp. *lactis* Bb-12	8.3 ^c^ ± 0.3	8.3 ^c^ ± 0.4	7.9 ^b,c^ ± 0.3	7.6 ^b^ ± 0.1	7.2 ^a,b^ ± 0.2	7.2 ^a,b^ ± 0.2	7.0 ^a^ ± 0.2	6.9 ^a^ ± 0.2

Legend: ^a,b,c,d,e^ Same letters in the same line indicate a lack of statistically significant difference between the means at the significance level *α* = 0.05.

**Table 6:** List of fermented white bean homogenate samples prepared as part of ice cream recipe.

Sample Code	Microbial Sample Composition
ics0	Control sample (nonfermented white bean homogenate)
ics1	White bean homogenate fermented by YC-180
ics2	White bean homogenate fermented by *L. acidophilus* La-5
ics3	White bean homogenate fermented by *L. plantarum* 299v
ics4	White bean homogenate fermented by *L. rhamnosus* GG
ics5	White bean homogenate fermented by *L. casei* DN-114001
ics6	White bean homogenate fermented by *B. animalis* subsp. *lactis* Bb-12

## Data Availability

The data supporting the findings of this study are available from the corresponding author (M.Z.) upon reasonable request.
